# Totally laparoscopic gastrectomy using intracorporeally stapler or hand-sewn anastomosis for gastric cancer: a single-center experience of 478 consecutive cases and outcomes

**DOI:** 10.1186/s12957-016-0868-7

**Published:** 2016-04-19

**Authors:** Ke Chen, Di Wu, Yu Pan, Jia-Qin Cai, Jia-Fei Yan, Ding-Wei Chen, Hendi Maher, Yi-Ping Mou

**Affiliations:** Department of General Surgery, Sir Run Run Shaw Hospital, School of Medicine, Zhejiang University, 3 East Qing Chun Road, Hangzhou, 310016 Zhejiang Province China

**Keywords:** Laparoscopic gastrectomy, Intracorporeal anastomosis, Stapling anastomosis, Hand-sewn, Stomach neoplasms

## Abstract

**Background:**

Totally laparoscopic gastrectomy (TLG) using intracorporeal anastomosis has gradually become mature thanks to the advancements of laparoscopic surgical instruments and the accumulation of operative experience. The goal of this study is to review our institution’s experience with TLG for the treatment of gastric cancer.

**Methods:**

A retrospective study was conducted to examine the short-term outcomes of TLG using intracorporeally stapler or hand-sewn anastomosis performed at Sir Run Run Shaw Hospital between March 2007 and June 2015. The details of intracorporeal anastomosis were described, and the clinicopathological data, surgical outcomes, and postoperative complications were evaluated.

**Results:**

Four hundred seventy-eight patients were included in the study. Generally speaking, the patients could be divided into stapler or hand-sewn groups according to whether intracorporeal anastomosis was performed by only hand-sewn technique (*n* = 97) or only stapling devices (*n* = 381). For overall patients, the mean operation time and anastomotic time were 225.7 and 30.0 min, respectively. Postoperative complications were observed in 65 patients. All of the patients recovered well without perioperative death by conservative or surgical management.

**Conclusions:**

TLG using intracorporeally stapler or hand-sewn anastomosis is a reasonable option for the treatment of gastric cancer, with early data showing acceptable perioperative outcomes.

## Background

Since the first case report of laparoscopic gastrectomy (LG) was reported in 1994, it has been used widely to treat gastric cancer due to the well-known short-term benefits, such as low rates of morbidities, decreased pain, shorter length of hospital stay, and less estimated blood loss [1–4].

In general, LG can be divided into laparoscopy-assisted and totally laparoscopic techniques. With laparoscopy-assisted gastrectomy (LAG), lymph node dissection is performed laparoscopically, but the transection of the stomach and the anastomosis are performed through an epigastric mini-laparotomy. Therefore, it may be difficult to perform the anastomosis through a small incision on patients with obesity with thick abdominal walls or on patients with a small remnant stomach due to poor visualization. This reconstructive modality might lead to pain and increased injury from the forceful traction at the mini-laparotomy site. It is reported that intracorporeal anastomosis with totally laparoscopic gastrectomy (TLG) have the advantages of safer anastomosis under better visualization, less postoperative adhesion, faster postoperative recovery, and smaller scars [5–7].

On the basis of our extensive laparoscopic experience gained from LAG, laparoscopic distal pancreatectomy, and other laparoscopic operations [8–11], we started to develop TLG for the treatment of gastric cancer and we initially used staplers to make intracorporeal anastomosis. However, in our practice, we have found some disadvantages of using staplers, especially for intracorporeal esophagojejunostomy. Therefore, we were encouraged to use the intracorporeal hand-sewn technique, mainly used for esophagojejunostomy after total gastrectomy. We report herein our experiences with the various types of anastomosis after TLG and also an evaluation of the postoperative surgical outcomes according to the type of anastomosis to assess those technical feasibilities and discuss the advantages as well as our experience.

## Methods

### Patients

We retrospectively analyzed a prospectively collected and maintained patient database. A total of 478 consecutive patients underwent TLG for gastric cancer between March 2007 and June 2015 in our department of Sir Run Run Shaw Hospital. Perioperative clinicopathological variables, such as gender, age, body mass index (BMI), preoperative physical classification defined by the American Society of Anesthesiologists (ASA) score, pathological diagnosis, tumor size, surgical records, and postoperative morbidity and mortality, were evaluated. Institutional Review Board approval was obtained before the initiation of this review.

### Surgical procedure

**Fig. 1:**
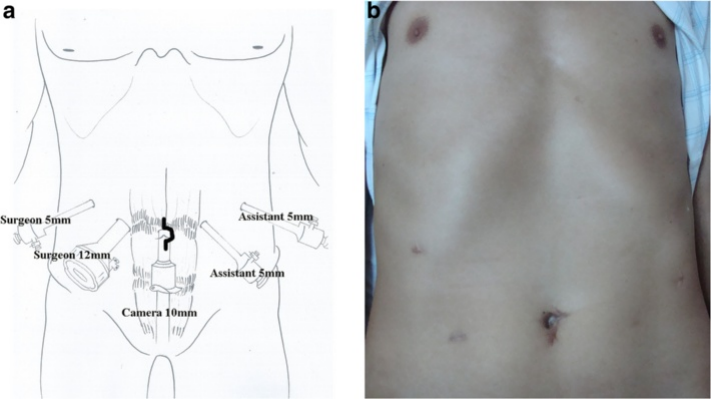
Trocar placement and incision. **a** Location of trocar placement and incision. **b** Postoperative view of the abdominal wound

Under general anesthesia, the patient was placed in supine position. Mobilization of the stomach and en bloc systematic lymph node dissection were performed via five trocars under a pneumoperitoneum (Fig. 1a). Sufficient lymphadenectomy is performed, and the stomach is transected. The resected specimen is removed through the extended umbilical incision, using a large plastic bag. An approximately 3–4-cm longitudinal incision was made to remove the specimen. The extended umbilical incision normally shrinks well within a few months (Fig. 1b). The detailed lymphadenectomy and resection procedure was described in our previously published articles [8–10].

**Fig. 2:**
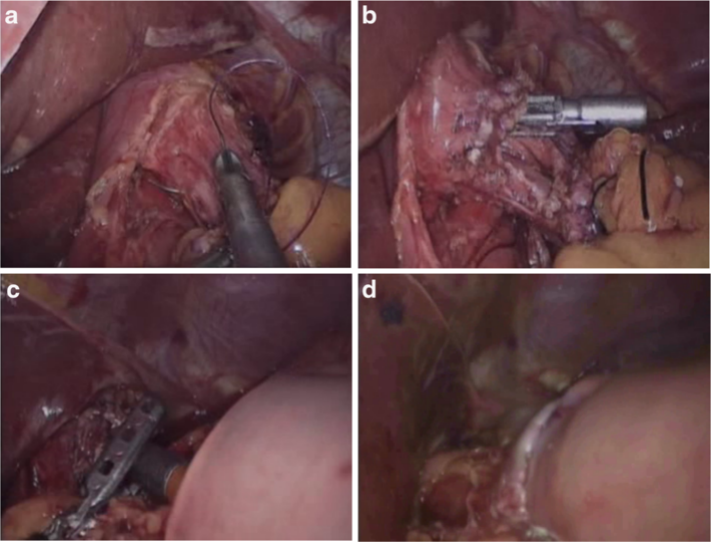
Intracorporeal conventional circular stapler-anvil end-to-side esophagojejunostomy. **a** The purse-string suture was placed on the esophagus. **b** The anvil was introduced into the esophageal stump. **c** The circular stapler was introduced into the jejunum through the jejunal stump and attached with the anvil. **d** The circular stapler fired and completed the esophagojejunostomy

**Fig. 3:**
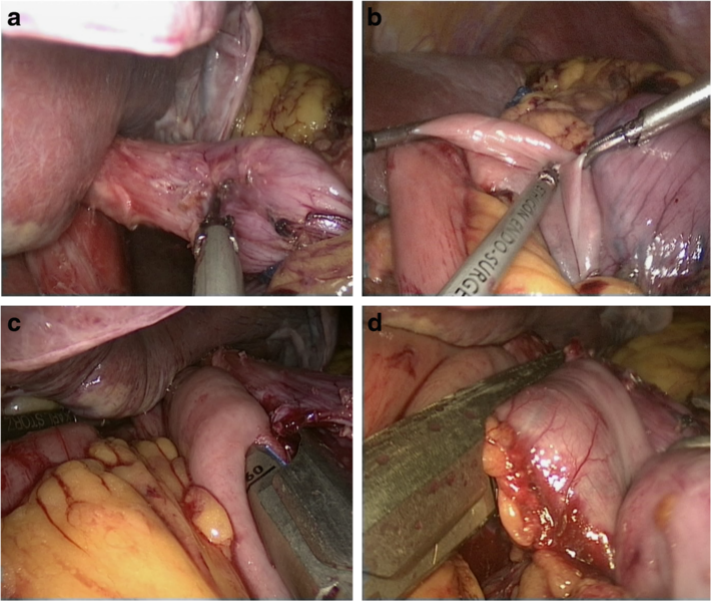
Intracorporeal linear stapler side-to-side esophagojejunostomy. **a** One hole was created on the posterior wall of the esophageal stump. **b** The other hole was created on the antimesenteric side of the efferent jejunal. **c** Each jaw of the linear stapler was inserted into the holes on the esophageal stump and the jejunum, and then, the linear stapler fired. **d** The entry hole and esophagus were closed using staplers
  - *Hand-sewn methods (type C):* The jejunal loop was brought up to reach the esophageal stump. The jejunum was anchored to the esophageal stump by several serosal muscularis interrupted sutures placed to the posterior layer of the esophageal stump (Fig. 4a). Two small holes were created: one on the antimesenteric side of the jejunum and the other on the esophageal stump (Fig. 4b). The posterior wall was closed by several full-thickness interrupted sutures (Fig. 4c), and closure of the anterior wall was carried out by a full-thickness continuous suture (Fig. 4d). The seromuscular layer was strengthened with interrupted sutures to reduce tension (Fig. 4e, f).

1. Methods of intracorporeal gastrointestinal reconstruction after total gastrectomy (Roux-en-Y)
  - *Mechanical stapler methods: conventional circular stapler-anvil method (type A):* The stomach was lifted up, and a purse-string suture was placed at 1 cm above the predetermined transected line (Fig. 2a). A hole was made at the esophagogastric junction using the Harmonic scalpel. The anvil was introduced into the esophageal stump through the hole, and the purse-string suture was tied (Fig. 2b). The esophagogastric junction was divided, and the stomach was extracted. The circular stapler was introduced into the jejunum through the jejunal stump (Fig. 2c). The circular stapler attached with the anvil and fired (Fig. 2d). The jejunal stump was closed with endoscopic linear staplers. *Linear stapler method (type B):* A small opening was made 10 cm from the stump on the distal jejunum (Fig. 3a), and the latter was then pulled up to the esophagus, in which a small side opening was also made (Fig. 3b). A side-to-side antiperistaltic esophagojejunostomy was then performed using linear staplers (Fig. 3c), and then, the entry hole and esophagus were closed using staplers (Fig. 3d).

**Fig. 4:**
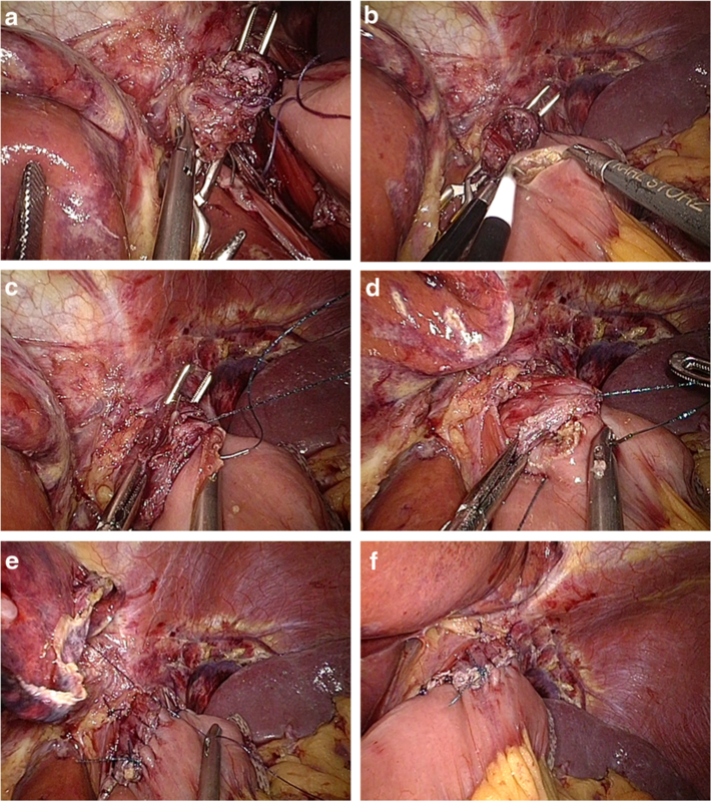
Intracorporeal hand-sewn end-to-side esophagojejunostomy. **a** The jejunum and esophageal stump attached to each other with seromuscular sutures. **b** A 2-cm-wide incision at the antimesenteric side of the jejunum. **c** Suture of the posterior wall using interrupted sutures. **d** Suture of the anterior wall using a continuous suture. **e** Strengthening of the seromuscular layer with interrupted sutures. **f** Complete esophagojejunostomy
2. Methods of intracorporeal gastrointestinal reconstruction after distal gastrectomy
  - *Mechanical stapler methods: linear stapler delta-shaped method (Billroth I, type D):* Small holes were then created along the edge of the gastric stump and duodenal stump (Fig. 5a). Then, they were approximated and joined with the endoscopic linear stapler (Fig. 5b). The staple line was then inspected for any defects, and hemostasis was verified. Stay sutures were placed to lift the common opening, which was then closed with two applications of the linear stapler (Fig. 5c, d). *Linear stapler side-to-side method (Billroth II, type E):* Two access openings were created: one on the posterior wall of the gastric stump 2 cm towards the cutting margin (Fig. 6a) and the other on the antimesenteric side of the efferent jejunal (15 cm distal to the ligament of Treitz) (Fig. 6b). One of the endoscopic linear stapler legs was inserted into the jejunum opening to draw the jejunum to the rear of the gastric stump. Then, the second leg was inserted into the stomach opening and fired (Fig. 6c). The common opening was closed with a continuous hand-sewn suture (Fig. 6d).

**Fig. 5:**
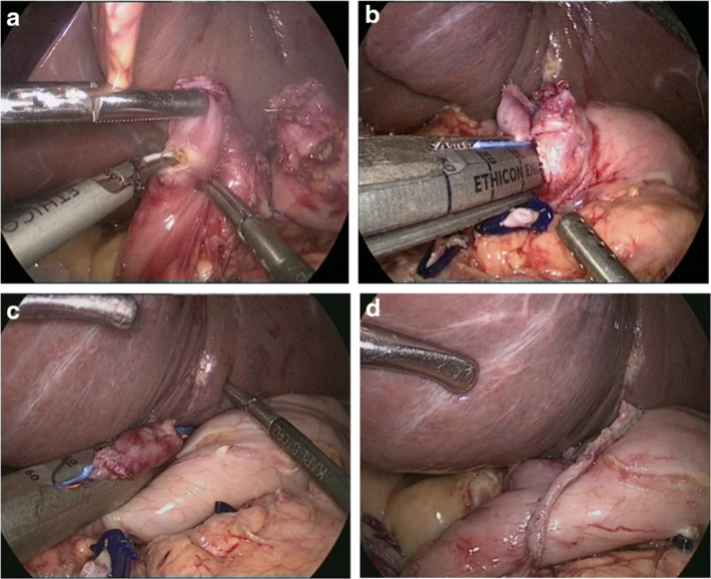
Linear stapler delta-shaped gastroduodenostomy. **a** Two small holes were created for jaw inserting. **b** The gastric stump and duodenal stump were approximated and joined with the endoscopic linear stapler. **c** The common opening was closed with two applications of the linear stapler. **d** Completed gastroduodenostomy

**Fig. 6:**
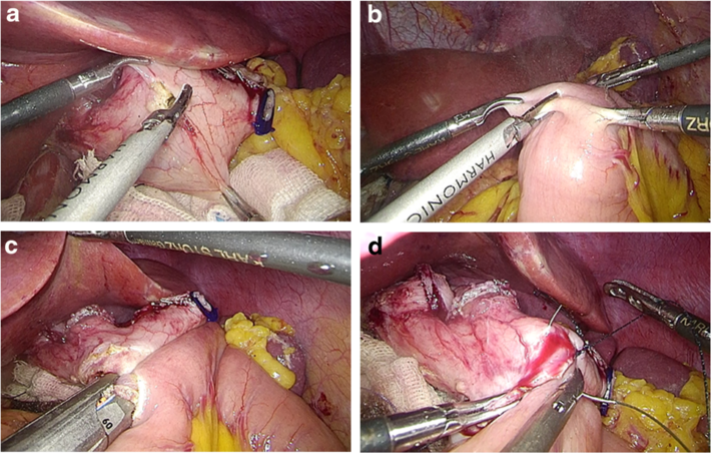
Linear stapler side-to-side gastrojejunostomy. **a** One hole was created on the posterior wall of the gastric stump. **b** The other hole was created on the antimesenteric side of the efferent jejunal. **c** Endoscopic linear stapler completing the anastomosis. **d** Laparoscopically closed common opening sewn by hand
  - *Hand-sewn methods: gastrojejunostomy (Roux-en-Y, type F):* A detachable laparoscopic intestinal clamp was placed at the greater curvature side of the gastric stump and transected with ultrasonic coagulating shears (Fig. 7a). The jejunal loop was introduced to approach the gastric stump. The details of hand-sewn gastrojejunostomy were similar to those of type C (Fig. 7b–d). Finally, a side-to-side jejunojejunostomy was performed through the enlarged umbilical incision. *Gastroduodenostomy (Billroth I, type G):* Two detachable laparoscopic intestinal clamps were placed at the pylorus and duodenum to avoid contamination. The duodenum was divided perpendicularly with ultrasonic coagulating shears between the two detachable clamps (Fig. 8a). The gastric stump was introduced to approach the duodenal stump. Then, several serosal muscularis interrupted sutures were made which are located at the rear part of the gastric and duodenal stump. A 3–4-cm-wide incision was made at the greater curvature side of the gastric stump for end-to-end gastroduodenostomy (Fig. 8b). The posterior wall of the esophagojejunostomy was sutured using interrupted sutures, and the anterior wall was sutured using a continuous suture (Fig. 8c). The seromuscular layer was strengthened with interrupted sutures to reduce tension (Fig. 8d). *Gastrojejunostomy (Billroth II, type H):* The jejunum loop 15 cm distal to the ligament of Treitz was introduced to approach the gastric stump. Then, several serosal muscularis interrupted sutures were made which are located at the rear part of the jejunum and gastric stump. A 3–4-cm-wide incision was made at the antimesenteric side of the jejunum for end-to-side gastrojejunostomy. The details of hand-sewn gastrojejunostomy were similar to those described above (Fig. 9).

**Fig. 7:**
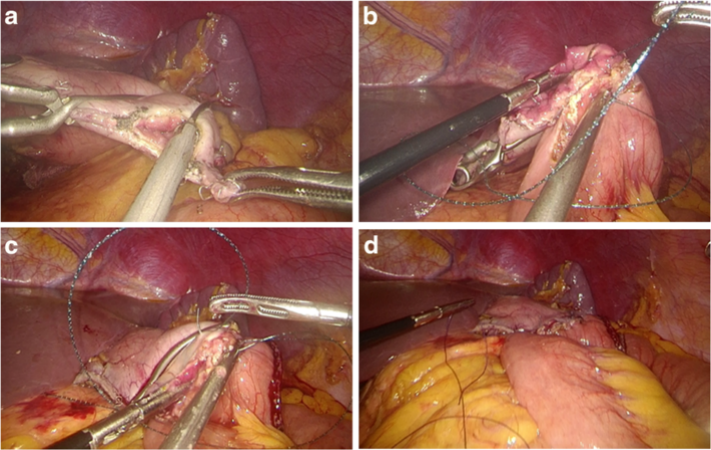
Intracorporeal hand-sewn end-to-side gastrojejunostomy. **a** Transection of the gastric stump with ultrasonic coagulating shears. **b** Suture of the posterior wall using interrupted sutures. **c** Suture of the anterior wall using a continuous suture. **d** Completed gastrojejunostomy

**Fig. 8:**
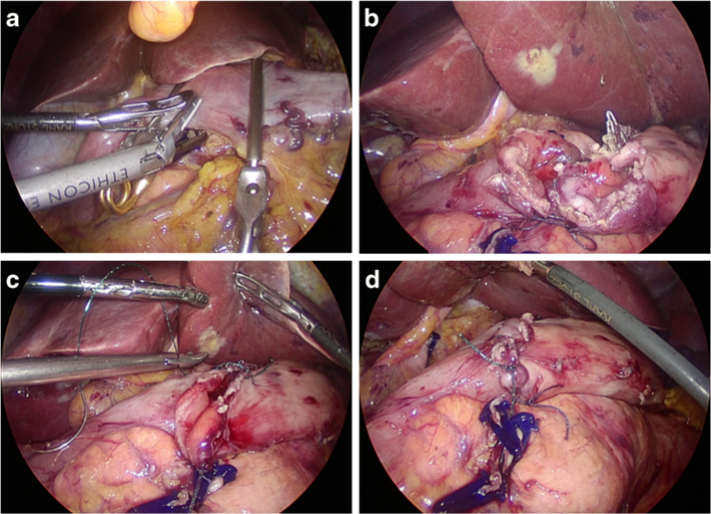
Intracorporeal hand-sewn end-to-end gastroduodenostomy. **a** Transection of the duodenum with ultrasonic coagulating shears between two clamps. **b** Ready for anastomosis after transection of the gastric stump. **c** Suture of the anterior wall using a continuous suture. **d** Completed gastroduodenostomy

**Fig. 9:**
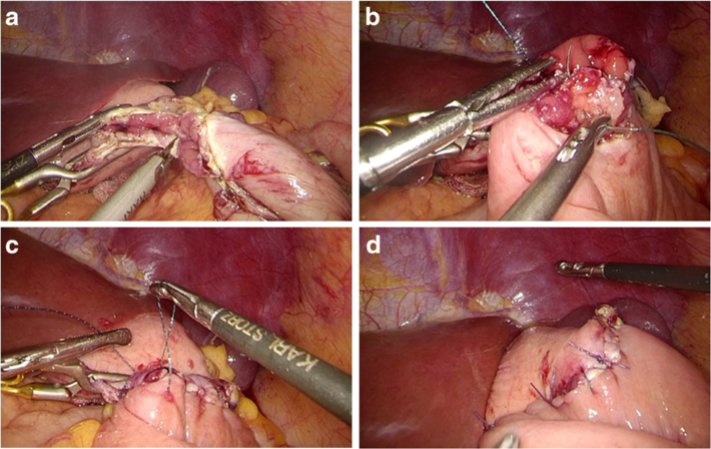
Intracorporeal hand-sewn end-to-side gastrojejunostomy. **a** Transection of the gastric stump with ultrasonic coagulating shears. **b** Suture of the posterior wall using interrupted sutures. **c** Suture of the anterior wall using a continuous suture. **d** Completed gastrojejunostomy

### Postoperative management

The nasogastric tube is removed immediately following the procedure, and all stable patients, without significant medical comorbidities, are transferred to the general ward for recovery. To be discharged from the hospital, patients had to adapt to a semiliquid diet, have a normal blood work panel and temperature, and not suffer from obvious discomfort.

## Results

### Demographic and clinicopathologic characteristics

The patient demographic and clinicopathologic characteristics are detailed in Table 1. Of the 478 patients, 99 underwent totally laparoscopic total gastrectomy (TLTG) and 379 underwent totally laparoscopic distal gastrectomy (TLDG). Two hundred sixty-five patients were men, and 213 patients were women. These patients had a mean age of 59.0 years and a body mass index of 23.0 kg/m^2^. Patients were classified as ASA I in 282 cases, ASA II in 162, and ASA III in 34. Analysis of pathologic tumor characteristics revealed that 168 patients had stage I tumors, 121 patients had stage II tumors, and 189 had stage III tumors.

**Table 1:** Clinicopathologic characteristics of patients

Variable	Total (*n* = 478)	TLTG (*n* = 99)	TLDG (*n* = 379)
Gender (male/female)	265/211	65/34	200/179
Age (years)	59.0 ± 12.0	52.0 ± 13.1	59.5 ± 11.0
BMI (kg/m^2^)	23.0 ± 3.1	22.9 ± 3.5	23.0 ± 2.9
ASA classification (I/II/III)	282/162/34	59/35/5	223/127/29
Comorbidities (yes)	75	23	52
Tumor size (cm)	4.0 ± 2.1	4.8 ± 2.5	3.9 ± 2.0
Histology (differentiated/undifferentiated)	289/189	64/35	225/154
TNM stage (I/II/III)	168/121/189	47/25/27	121/96/162

Data are means ± standard deviations or number (%)

*BMI* body mass index, *ASA* American Society of Anesthesiologists

### Operative findings and postoperative clinical course

The operative findings and subsequent postoperative clinical course data are shown in Table 2. No procedures were converted to open or other laparoscopic anastomosis techniques. TLTG with mechanical stapler intracorporeal anastomosis was used for 40 patients (type A 12 and type B 18) and hand-sewn for 59 patients (type C 59). In the TLTG group, the operation time was 257.4 ± 47.2 min for the hand-sewn procedure and 284.3 ± 45.6 min for the stapler procedure. The average time of intracorporeal hand-sewn esophagojejunostomy was 46.3 ± 10.8 min and 47.9 ± 17.1 min for the stapler one. The mean times to first flatus, start diets, and postoperative hospital stay were 3.9 days (range, 2–7 days), 5.1 days (range, 3–7 days), and 10.5 days (range, 8–20 days), respectively, for intracorporeal stapler esophagojejunostomy and 3.7 days (range, 2–6 days), 4.8 days (range, 3–8 days), and 9.4 days (range, 6–22 days), respectively, for intracorporeal hand-sewn esophagojejunostomy.

**Table 2:** Operative findings and postoperative clinical course

	TLTG (*n* = 99)		TLDG (*n* = 379)		Total (*n* = 478)
	Stapler (*n* = 40)	Hand-sewn (*n* = 59)	Stapler (*n* = 341)	Hand-sewn (*n* = 38)	
Operation time (min)	284.3 ± 45.6 (230–380)	257.4 ± 47.2 (170–350)	213.8 ± 45.1(120–360)	221.4 ± 26.8 (180–280)	225.7 ± 49.6 (120–380)
Anastomotic time (min)	47.9 ± 17.1(25–90)	46.3 ± 10.8 (29–67)	23.1 ± 5.1 (13–45)	33.5 ± 7.7 (21–50)	30.0 ± 14.0 (13–90)
Blood loss (mL)	83.8 ± 35.2 (30–200)	87.6 ± 42.4 (30–200)	101.4 ± 69.5 (10–400)	94.7 ± 30.3 (50–150)	97.7 ± 62.2 (10–400)
Retrieved lymph nodes	32.9 ± 5.3 (24–45)	38.9 ± 13.4 (25–42)	32.6 ± 9.7 (9–81)	30.8 ± 8.4 (21–55)	33.2 ± 10.1 (9–81)
First flatus (days)	3.9 ± 1.1 (2–7)	3.7 ± 1.0 (2–6)	3.7 ± 1.2 (1–9)	3.3 ± 0.8 (2–5)	3.7 ± 1.1 (1–9)
Liquid diet (days)	5.1 ± 1.0 (3–7)	4.8 ± 1.2 (3–8)	4.7 ± 2.0 (2–18)	4.4 ± 0.9 (3–7)	4.7 ± 1.8 (2–18)
Soft diet (days)	6.5 ± 1.2 (4–11)	6.6 ± 1.5 (4–12)	6.6 ± 2.6 (3–20)	6.4 ± 1.2 (4–9)	6.6 ± 2.3 (3–20)
Postoperative hospital stay (days)	10.5 ± 2.5 (8–20)	9.4 ± 2.9 (6–22)	9.6 ± 3.4 (4–23)	9.3 ± 1.7 (7–13)	9.6 ± 3.2 (4–23)

Data are means ± standard deviations (range)

The types of anastomotic methods in TLDG were mechanical staplers in 341 patients (type D 23 and type E 318) and hand-sewn in 38 patients (type F 16, type G 14, and type H 8). The mean operation time and intracorporeal anastomosis time were 213.8 min (range, 120–360 min) and 23.1 min (range, 13–45 min), respectively, for mechanical stapler intracorporeal anastomosis and 221.4 min (range, 180–280 min) and 33.5 min (range, 21–50 min), respectively, for hand-sewn anastomosis. The mean times to first flatus, start diets, and postoperative hospital stay were 3.7 days (range, 1–9 days), 4.7 days (range, 2–18 days), and 9.6 days (range, 4–23 days), respectively, for intracorporeal stapler anastomosis and 3.3 days (range, 2–5 days), 4.4 days (range, 3–7 days), and 9.3 days (range, 7–13 days), respectively, for intracorporeal hand-sewn anastomosis.

### Postoperative complications

The postoperative complications are listed in Table 3. There were no in-hospital mortality and 30-day mortality. Complications developed in 16 (16.2 %) of patients in the TLTG group and 49 (12.9 %) of patients in the TLDG group. Six patients in the TLTG group had anastomotic complication regarding leakage, stricture, and intraluminal bleeding. Twelve patients in the TLDG group had anastomotic complication, two for anastomotic leakage, three for anastomotic stricture, and seven for intraluminal bleeding.

**Table 3:** Postoperative complications

	TLTG (*n* = 99)		TLDG (*n* = 379)		Total (*n* = 76)
	Stapler (*n* = 40)	Hand-sewn (*n* = 59)	Stapler (*n* = 341)	Hand-sewn (*n* = 38)	
Postoperative complications	10	6	39	10	65
Anastomotic leakage	1		2		3
Anastomosis stricture	3		1	2	6
Intraluminal bleeding	1	1	4	3	9
Delayed gastric emptying	1		5	1	7
Abdominal abscess	1	2	8	1	12
Ileus			6	1	7
Lymphorrhea	1		2		3
Pancreatic leakage		2	4		6
Pulmonary infection	1	1	7	2	11
Pulmonary embolism	1				1

## Discussion

The most popular version of LG is LAG, wherein the lymph node dissection is completed under the laparoscope. Then, the extracorporeal anastomosis with LAG was performed through a 50–70-mm small incision in the middle upper abdomen. Performing the anastomosis in this narrow and restricted space is often difficult, especially on obese patients with thick abdominal walls or on patients with a small remnant stomach. It should be noted that the inclusion of the auxiliary incision in LAG makes it divergent from the minimally invasive treatment concept pursued in laparoscopic surgery. Previous studies reported some advantages of TLG over LAG such as better cosmesis, less blood loss, and faster recovery. And as our essays issued before [8], in practice, we have found that TLG is preferable to LAG for three additional reasons. First, TLG enables a tension-free anastomosis and thus avoids damage to the surrounding structures. Second, TLG is more suitable for a “no touch tumor” operation. Finally, TLG requires only a small incision and imparts more selectivity to the surgeon than LAG. However, until now, LAG is still the most commonly performed type of LG [12]. The development of TLG has been limited because successful reconstruction of the digestive tract laparoscopically has been difficult to achieve, especially for intracorporeal esophagojejunostomy. Hence, there is a need to develop a more standardized methodology in reconstructing the digestive tract by the laparoscopic approach that is as simple and safe as possible.

The methods of gastrointestinal anastomosis after laparoscopic distal gastrectomy (LDG) are the same as those of standard laparotomy which include the Billroth I, Billroth II, and Roux-en-Y methods. All of the methods are safe and efficacious; however, there have been no statistically significant differences in the early postoperative outcomes among the three reconstruction methods [13–15]. The ideal gastrointestinal reconstruction procedure should minimize postoperative morbidity and improve quality of life [16]. Billroth I and Roux-en-Y procedures are the commonly used reconstruction techniques following resection of open distal gastrectomy (DG). Billroth I reconstruction has commonly been employed after DG for gastric cancer due to its simplicity, physiological advantage of allowing food to pass through the duodenum, and ease of postoperative endoscopy allowing access to the papilla of Vater [17, 18]. However, there are three most common drawbacks of the Billroth I anastomosis, remnant gastritis, reflux esophagitis, and limitation in extent of resection.

Traditionally, Roux-en-Y reconstruction has been the reconstruction method of choice in total gastrectomy (TG) [18] and is being increasingly used to prevent duodenogastric and gastroesophageal reflux in DG [19–21]. However, Roux-en-Y gastrojejunostomies have their disadvantages as follows: ulcerogenic and Roux stasis syndrome [22]. Moreover, it is complex, technically difficult, and time consuming, resulting in prolonged operative time under the totally laparoscopic intracorporeal procedure. And if not hand-sewn, the extensive use of endoscopic linear staplers can result in higher costs [23]. Therefore, Roux-en-Y reconstruction is commonly performed extracorporeally through a mini-laparotomy incision in LAG.

Billroth II after DG is an alternative for reconstruction of the alimentary tract when Billroth I and Roux-en-Y reconstructions are difficult or unrealistic. The merits of Billroth II reconstruction compared to Billroth I are a lower food stasis rate and a larger extent of resection. If the tumor is located in the middle third of the stomach, it is difficult to perform Billroth I reconstruction because excessive tension might develop at the anastomosis site if a safety margin was included. And in China, the most gastric cancer cases are advanced stage, which need more radical resection. Based on the fact above, the most commonly used intracorporeal anastomosis method in DG in our center is Billroth II reconstruction. If Roux-en-Y reconstruction was used, we only choose the hand-sewn approach, which is more economical.

Regarding intracorporeal linear stapler side-to-side Billroth II reconstruction, we have summarized three points of experience as follows [8]: First, anastomosis should be made at the posterior wall of the remnant stomach parallel to the greater curvature. Second is using a stapler to make position of the jejunum and gastric stump directly, instead of fixed by them using stitches before staples are applied. Third is using a manual continuous suture to close the common opening, instead of endoscopic linear staplers. It is reported that the delta-shaped anastomosis is a simple, easy, and safe method of intracorporeal gastroduodenostomy [24]. We also used it for intracorporeal end-to-end Billroth I reconstruction and summarized three main points as follows: (1) Three stay sutures were placed to each end of the common opening and cutting edges of the stomach and duodenum to achieve a better involution. (2) The liner stapler is better to be vertical to the cutting edges of the stomach and duodenum. (3) Two steps are recommended during the closure of the common opening which is likely to avoid the anastomosis stricture.

For intracorporeal mechanical esophagojejunostomy in TG, the first 18 patients in our series used the conventional circular stapler-anvil method. Based on our experience, the esophagus was not cut off at first while the cardia was tightly tied with a band and then stretched down to well expose the esophagus. Purse-string suture was performed, and then, the anterior wall of the esophagus was cut with the Harmonic scalpel for a half-circle. After placement of the anvil, the suture line was tightened and the esophagus was finally cut off with the Harmonic scalpel. However, the circular stapler was inappropriate for placement during laparoscopic surgery due to its bigger size and absence of matching tube. The pneumoperitoneum was vulnerable to its placement, and the vision is unclear. The inserted anvil (OrVilTM; Covidien Mansfield, MA, USA) was introduced to simplify the procedure of anvil placement, which was reported safe and effective [25]. However, because of its high cost, possibility of bacterial contamination in the abdominal cavity, and injury of the esophageal mucous, we did not use this method.

The linear stapler side-to-side method was simple in operation, and the anastomotic stoma was bigger, which can avoid the postoperative complications such as anastomotic stenosis. For position of the jejunum and esophageal stump, like linear stapler side-to-side Billroth II reconstruction, we used a stapler to make position directly. However, there are possible problems in this method, such as distortion of the Roux limb or mesenterium and slipping of the esophagojejunal anastomotic site into the lower mediastinum. The surgical margin is limited for longer esophageal stump should be reserved.

Hand-sewn end-to-side esophagojejunostomy overcomes the limitations caused by the mechanical method. This method completes the anastomosis after removal of the specimen. The anastomosis can be performed after intraoperative frozen section evaluation and confirmation of negative margins. And this method does not need longer esophageal stump. For patients with positive resection margin, the removal extent can be expanded appropriately to confirm negative resection margin. However, the hand-sewn method requires the operators with rich experience in laparoscopic suture skill, and it takes longer time. According to our experience, progressive practice can effectively shorten the learning curve. At the same time, the application of some new laparoscopic instruments can simplify the intracorporeal hand-sewn suture.

We recommend that the reconstruction method using a stapler should be selected on the basis of the location of the tumor. Our experience is that the side-to-side esophagojejunostomy using a linear stapler can be adopted for patients with lesions in the body and fundus of the stomach as well as the lower cardia. For patients with lesions in the upper and middle cardia, end-to-side esophagojejunostomy using a circular stapler is still chosen for enough surgical margins. Also, if the surgeon was well experienced with the laparoscopic hand-sewn technique, it can be used after TG regardless of tumor location.

The hand-sewn technique is quite difficult but not impossible. We are very willing to provide some experienced tips: First, knotless barbed sutures (V-LocTM; Covidien Mansfield, MA, USA) are recommended. It can shorten the time of anastomosis and can ensure the safety of anastomosis, with no need for permanent traction during the whole anastomosis process. Second, keep two long corner stay sutures respectively at the 3 o’clock and 9 o’clock positions of the anastomotic stoma when you are performing the posterior wall anastomosis, which is the most challenging step. This tip is able to maintain tension to provide a clear view of the posterior wall and allow more precise anastomosis. Maintaining the integrity of this anastomosis is important, and lessening the tension of the anastomosis is also a key point in preventing the occurrence of bile leakage. Moreover, interrupted sutures of the seromuscular layer are also helpful to reduce tension. The specially developed laparoscopic clamps play a crucial part in the success of the techniques. The clamps prevent fecal contamination of the abdominal cavity and facilitate the performance of the anastomosis.

## Conclusions

Our results demonstrated that TLG with intracorporeal anastomosis is a feasible procedure that can be safely performed. Linear stapler side-to-side Billroth II reconstruction may be a simple and less time-consuming procedure after DG if the delta-shaped Billroth I reconstruction cannot be used. For intracorporeal esophagojejunostomy after TG, the mechanical methods can also be safely performed with the proper experience. However, there may be some limitations. Therefore, the hand-sewn method is recommended if the surgeons are familiar with the intracorporeal hand-sewn suturing technique.

## Institutional review board statement

The study was reviewed and approved by the Zhejiang University Institutional Review Board.

## Informed consent statement

All study participants, or their legal guardian, provided informed written consent prior to study enrollment.
